# Adenovirus as a Vector and Oncolytic Virus

**DOI:** 10.3390/cimb45060307

**Published:** 2023-06-02

**Authors:** Wataru Matsunaga, Akinobu Gotoh

**Affiliations:** 1 Joint-Use Research Facilities, Hyogo Medical University, 1-1 Mukogawa, Nishinomiya 663-8501, Japan; 2 Department of Education for Medical Research Base, Hyogo Medical University, 1-1 Mukogawa, Nishinomiya 663-8501, Japan

**Keywords:** adenovirus, adenoviral vector, oncolytic virus, midkine

## Abstract

Adenoviral vectors, both oncolytic viruses and gene delivery vectors, are among the earliest approved and commercialised vectors for gene therapy. Adenoviruses have high cytotoxicity and immunogenicity. Therefore, lentiviruses or adeno-associated viruses as viral vectors and herpes simplex virus as an oncolytic virus have recently drawn attention. Thus, adenoviral vectors are often considered relatively obsolete. However, their high cargo limit and transduction efficiency are significant advantages over newer viral vectors. This review provides an overview of the new-generation adenoviral vectors. In addition, we describe the modification of the fiber knob region that enhances affinity of adenoviral vectors for cancer cells and the utilisation of cancer-cell-specific promoters to suppress expression of unwanted transgenes in non-malignant tissues.

## 1. Introduction

Adenoviruses are DNA viruses with non-enveloped icosahedral particles that are approximately 90 nm in diameter. Currently, 88 types of human adenoviruses (1–88) have been identified and classified into seven serotypes (A–G) [1], many of which cause various diseases in humans. However, in 1993, gene transfer into neurons using adenoviral vectors was reported [2], whereafter adenoviruses began to attract attention as efficient vectors that could also transfer genes into non-dividing cells. Despite being one of the most common viral human pathogens, adenoviruses are also among the most widely used viral vectors in gene therapy research. Adenoviral vector use accounts for 15.5% of vector-based gene therapy clinical trials worldwide [3]. Moreover, adenoviruses have attracted attention because they have been used as a base material for viral vector vaccines against coronavirus disease 2019 [4].

Currently, 68.2% of clinical gene therapy trials are aimed at cancer treatment [3]. Virotherapy for cancer uses a genetically modified virus to introduce a therapeutic gene as a vector or uses it as an oncolytic virus. In recent years, viral immunotherapy has been widely studied.

Adenoviral vectors used in gene therapy research, including studies on virotherapy, are mostly based on adenovirus serotype 5 (Ad5). This is possibly because Ad5 was the first to be analysed for gene function, knowledge about Ad5 has already been accumulated, and high-titer viral particles can be produced easily. However, studies using lentiviruses and adeno-associated viruses (AAV) as subjects for virus therapy have recently increased, and when compared with these “newcomer” viral vectors, some researchers consider adenoviral vectors “obsolete”.

In this article, we briefly describe the characteristics of adenoviruses as vectors or oncolytic viruses and the present status of studies using them.

## 2. Trends of Viral Vectors

Currently, the top five vectors used in gene therapy clinical trials worldwide are adenoviruses, retroviruses, plasmids, lentiviral vectors, and AAV vectors [3]. Adenoviral vectors are among the earliest vectors used in the history of gene therapy and, as of 2022, will account for the largest share of vectors used in gene therapy clinical trials worldwide (Table 1). Although adenoviruses still rank first in terms of the percentage of total cases in which they are used, the share of adenoviral vectors has declined over the past 10 years [5,6]. However, the use of lentiviral and AAV vectors has significantly increased. Comparing the number of cases from 2012 to 2017, lentiviral vectors ranked first and adenoviral vectors third, whereas those from 2017 to 2022 showed adenovirus vectors ranking the lowest among all vectors listed in Table 1. Considering this, adenoviruses appear to be “obsolete” vectors. However, they still offer several advantages. In addition, among the many serotypes of adenoviruses known, it is conceivable that a completely new adenoviral vector suitable for human clinical applications may be discovered.

Characteristics of the top five vectors used in gene therapy clinical trials are shown in Table 2.

Adenoviruses, which accounted for the largest share of clinical trials, are the main subject of this article and will be discussed in the next chapter. Below is a summary of the other viral vectors listed in Table 1 and Table 2.

### 2.1. Retroviral Vectors

Retroviral vectors generally refer to vectors derived mainly from the Moloney murine leukaemia virus, a type of gammaretrovirus, and are distinguished from lentiviral vectors, which are retroviruses. Retroviral vectors are commonly used in gene therapy because of their low cytotoxicity. Long-term stable gene expression is also an advantage of retroviral vectors; however, their inability to transfer genes into non-dividing cells is a major drawback. Moreover, the potential problems of mutagenesis randomly affecting chromosomes and activating nearby proto-oncogenes to induce cancer is not negligible [7,8]. The retroviral vector cargo limit of 9–10 kb is moderately large but inferior to that of new-generation adenoviral vectors.

### 2.2. Lentiviral Vectors

Lentiviral vectors are a subtype of retroviral vectors developed based on HIV-1 and are capable of transducing genes into almost all mammalian cells, including non-dividing cells that cannot be transducted with retroviral vectors. Similar to retroviral vectors, lentiviral vectors can maintain stable gene expression. Lentiviral vectors have been widely used to study ex vivo cancer immunotherapy via T cells using chimeric antigen receptors [9]. As a result of improvements made to enhance safety, third-generation lentiviruses have acquired low immunogenicity and cytotoxicity. However, because lentiviral vectors are based on HIV-1, some HIV-1 genes are required for vector production, and safety concerns regarding their use in gene therapy remain. In addition, lentiviruses cannot be used as oncolytic viruses because they lack replication ability. Their cargo limit of 9–10 kb seems sufficient, but it is still inferior to that of new-generation adenoviruses.

### 2.3. Adeno-Associated Virus (AAV) Vectors

AAVs are single-stranded DNA parvoviruses that can replicate only in the presence of other “helper viruses”, such as the adenovirus or human papillomavirus. Eleven AAV serotypes (1–11) have been identified [10], which differ in their tropism to the types of cells they infect. Therefore, AAV vectors are useful for the preferential transduction of specific cell types. AAV2 is the most well-characterised and most used AAV vector. Although AAV immunogenicity and high-dose AAV-vector-induced hepatotoxicity have been frequently reported [11], there are no known infections caused by AAV in humans, and its pathogenicity is thought to be almost negligible. Taken together, AAV vectors, which are safe and have a high transduction efficiency, are promising viral vectors. Studies on gene therapy using AAV vectors include the treatment of various intractable diseases, such as haemophilia [12] and Parkinson’s disease [13]. Regarding cancer treatment, a genetically engineered AAV vector that selectively binds to the tumour antigen, Her2/neu, and can only infect cancer cells has been reported [14]. Moreover, Onasemnogene abeparvovec (Zolgensma), an AAV9-based gene therapy drug used to treat spinal muscular atrophy, has been marketed. This drug is very expensive but shows remarkable therapeutic efficacy [15,16].

However, AAV exhibits distinct drawbacks. The cargo limit of AAV (4.7–5 kb) is too small to accommodate the large sizes of therapeutic genes that may occur with the development of gene therapy in the future. AAV vectors allow for the stable expression of transducted genes; however, due to various factors, stable expression of transgenes is not always possible. Furthermore, purification of high-titer AAV vectors is relatively difficult.

## 3. Adenovirus as a Vector

The advantages of adenoviral vectors include a large cargo limit and high efficiency of infection. High cytotoxicity is a major drawback of adenoviral vectors; however, when used as oncolytic viruses, this trait proves to be powerful and advantageous. Transient gene expression is also a drawback, but this leads to better safety because gene expression would not last long when acting on unintended targets.

### 3.1. First-Generation Adenoviral Vectors

**Figure 1:**
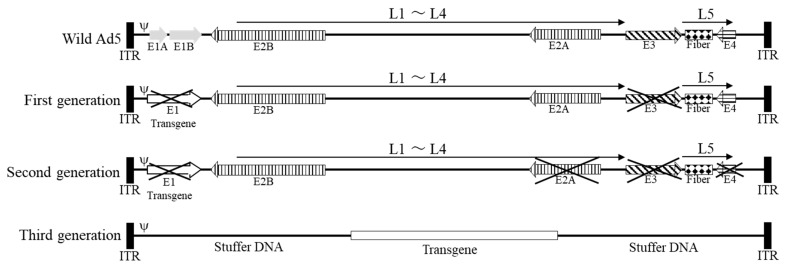
Genomic overview of each generation of adenoviral vectors. E1~E4, early regions 1–4; L1~L5, late regions 1–5; Ad5, adenovirus serotype 5; ITR, inverted terminal repeat; ψ, packaging signal.

Adenoviral vectors are classified according to their genetic design, from first to third generation. First-generation adenoviral vectors were designed to delete the Early 1 (E1) and Early 3 (E3) regions (Figure 1). The E1 region encodes genetic information essential for adenovirus survival and replication [17]; however, this region is deleted in first-generation adenoviruses because it prevents uncontrolled adenoviral replication and can be replaced with the contents of packaging cells [18]. The E3 region encodes a protein that protects infected cells against the immune response [19]. It is often deleted in the first generation to increase the cargo capacity because it is not essential for replication. By deleting E1 and E3, 5–6 kbp of cargo capacity can be secured. First-generation adenoviral vectors are incapable of self-replication and require a packaging cell line expressing E1 protein for their production. Human embryonic kidney 293 cells are the most commonly used packaging cells [18].

### 3.2. Second-Generation Adenoviral Vectors

Because several human cells express E1A-like factors [20], even first-generation adenoviral vectors with deleted E1 regions can induce potent host immune responses and chronic cytotoxicity in transducted host cells [21]. A second-generation adenovirus with E2 and E4 deletions was developed to attenuate the host cell immune response to the vector (Figure 1). The E2 region contains genes required for adenovirus replication [22], and the E4 region encodes regulatory proteins for DNA transcription [23]. Deleting these regions provides approximately 14 kb free space and significantly reduces native adenovirus protein synthesis. Nevertheless, second-generation adenoviruses still induce host immune responses through adenoviral proteins expressed by the remaining genes, resulting in reduced transgene expression in target cells [24].

### 3.3. Third-Generation Adenoviral Vectors

Despite deletion of the E1–E4 regions, first- and second-generation adenoviral vectors still have considerable immunogenicity and cytotoxicity. In addition, at the beginning of the 21st century, genes exceeding the capacity limit of conventional adenoviral vectors were considered therapeutic genes; therefore, third-generation adenoviral vectors were developed [25,26]. Third-generation adenoviral vectors have their entire native adenoviral genome removed, except for the inverted terminal repeat and ψ packaging signal, thereby increasing the cargo limit to 36 kb. This third-generation feature, also called a “gutless adenovirus vector”, allows high-level expression in host cells with no expression of viral proteins and little induction of an immune response [27].

Owing to the lack of genes required for self-assembly, a “helper adenovirus” carrying the genes required for replication must be co-introduced into the packaging cells when producing third-generation adenoviral vectors, which is also called a “helper-dependent vector”. However, there are concerns that helper adenoviruses will contaminate the final product of third-generation adenoviruses and that homologous recombination between helper viruses and packaging cells will result in self-propagating adenoviruses. Therefore, genetic modifications of adenoviruses used as helper viruses or methods using non-adenoviruses as helpers have been investigated [28]; however, viral approaches have not resolved the concern of contamination. Nevertheless, a contamination-free third-generation adenoviral vector production method has been developed that uses plasmids encoding the necessary genes as “helpers” [28,29].

For third-generation adenoviruses, it is necessary to place other genes (stuffer DNA) in regions other than the target gene for efficient encapsidation [30]. However, the effect of stuffer DNA on vector gene transfer efficiency remains unclear. Some reports state that stuffer DNA affects transduction efficiency [31,32], whereas others claim that it does not [33].

### 3.4. Current Status of Adenoviral Vectors

At present (June 2023), Nadofaragene firadenovec (brand name: Adstiladrin, also known as rAd-IFNa/Syn3) is the only approved adenovirus vector for gene therapy. Nadofaragene firadenovec is an E1-deleted, replication-deficient adenoviral vector based on Ad5 that contains a human IFN-α2b gene [34,35]. It was approved in the United States for the treatment of non-muscle-invasive bladder cancer (NMIBC) in December 2022 [36]. Currently, most clinical trials using non-proliferating adenovirus vectors are vaccine studies for COVID19. Meanwhile, in cancer therapy research, trials using adenoviral thymidine kinase (ADV-Tk) to treat hepatocellular carcinoma are in phase III (NCT03313596) [37]. 

## 4. Adenovirus as an Oncolytic Virus

Surgery, radiation, and anticancer agents are the three pillars of cancer therapy, and cancer immunotherapy has recently been included as a standard cancer therapy. However, surgery places a heavy burden on the body and is not always feasible for all patients with cancer. Other standard treatments have side effects that significantly impair patients’ quality of life (QOL). In this regard, oncolytic virus therapy has attracted attention as a therapeutic method that causes little deterioration in QOL.

The basic concept of an oncolytic virus is that it preferentially infects and proliferates in cancer cells, thereby lysing and destroying them. The virus released at that time also infects surrounding cancer cells, causing a chain of destruction in tumour tissues [38]. The origin of the concept of oncolytic viruses dates back to the discovery of viruses. In the late nineteenth and early twentieth centuries, when viruses began to be discovered, a relationship between temporary cancer remission and viral infections was reported [39,40,41].

In the 1920s, viral infection-induced oncolysis was confirmed in animal experiments. From the late 1940s to the 1960s, clinical studies of oncolytic virotherapy using wild-type viruses, such as yellow fever or West Nile viruses, were extensively conducted, but the results of these studies indicated a lack of safety or efficacy. Therefore, studies of oncolytic virus therapy were abandoned [39,40,41]. However, with the establishment of genetic engineering technology in the 1990s, highly safe viruses that replicate only in cancer cells have become a reality, and oncolytic virus therapy has once again become the focus of attention.

In 1997, the first oncolytic virus, named ONYX-015, was reported [42]. This oncolytic virus is an adenovirus lacking E1B 55-Kilodalton-Associated Protein, which inactivates the tumour suppressor gene p53 [43] and selectively infects and lyses human cancer cells in which the p53 gene is non-functional.

A clinical trial was conducted to treat head and neck cancer using ONYX-015 [44]. However, ONYX-015 must be injected directly into the tumour because it is highly toxic when administered intravenously. Therefore, it could only be applied to large tumours. Because it did not show the expected antitumour effect, the ONYX-015 project was aborted in 2000 [45]. However, the H101 strain (Oncorine), a genetically modified adenovirus based on ONYX-015, was approved by the Food and Drug Administration of the People’s Republic of China in 2005 for treating head and neck cancer [43]. It is the first approved oncolytic virus worldwide, aside from Rigvir, which has unclear efficacy, and has been approved in the Republic of Latvia [46].

Recently, the herpes simplex virus seems to have attracted more attention than the adenovirus as an oncolytic virus, and T-VEC, G47Δ (Teserpaturev) [47,48] and C-REV (originally HF10) [49] have already been approved or are in clinical trials. Moreover, novel adenovirus-based oncolytic viruses have also been investigated. At present, OBP-301 (suratadenotureb) [50,51] is undergoing a phase II clinical trial [52] for the treatment of head and neck cancer (NCT04685499), and CG0070 Adenoviral vector is phase II/III (NCT01438112) [53]. 

Although oncolytic viruses have increased infection specificity for cancer cells, they are still capable of infecting non-malignant cells. Therefore, the development of new-generation oncolytic viruses requires further improvements to enhance their specificity. For adenovirus-based oncolytic viruses, replacement of the Ad5 fiber knob with other adenovirus serotypes has been shown to increase the infectivity of cancer cells. Initially, a vector was developed in which the adenovirus type 35 (Ad35) fiber knob region was introduced into Ad5. Currently, Ad3 and Ad37 fiber knobs are also being studied [54]. 

Ad5 is transduced into cells through the coxsackie and adenovirus receptor (CAR) [55]. However, CAR expression is generally low in cancer cells [56,57], although its upregulation has been reported in some of them [57]. Therefore, genetic modification of the fiber knob region, where the adenovirus binds to its receptor, is the main method used to resolve the reduced efficiency of CAR-mediated gene transfer to cancer cells.

**Figure 2:**
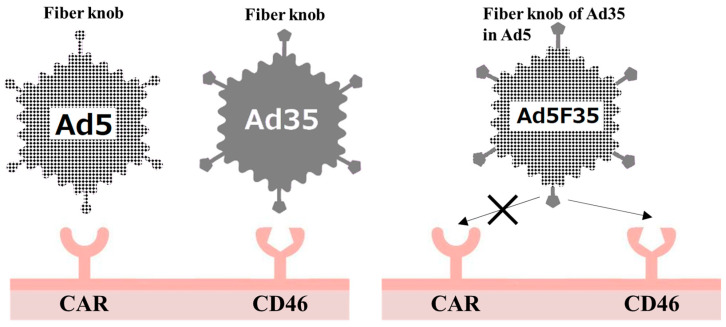
Concept of the chimeric adenoviral vector, adenovirus serotype 5 F35 (Ad5F35). Ad5 infects cells via the coxsackie and adenovirus receptor (CAR), but CAR is poorly expressed in cancer cells. Ad35 infects via ubiquitously expressed cluster of differentiation 46 (CD46). Ad5 and Ad35 have no affinity for CD46 and CAR, respectively. Therefore, when the fiber knob region of Ad5 is converted to that of Ad35, it is possible for adenoviral vectors to infect via CD46.

Cluster of differentiation 46 (CD46), which acts as a receptor for serotype B adenoviruses [58], is expressed in almost every human cell [58,59]. Ad35, which belongs to serotype B, does not interact with CAR, and the adenovirus serotype 5 F35 (Ad5F35) vector, in which the fiber knob region of the Ad5 vector is replaced with that derived from Ad35, binds to CD46 (Figure 2). Thus, it is effective in cells lacking CAR [60,61]. Several groups have investigated the utility of Ad5F35 in the development of transduction efficacy [62,63,64]. In 2021, it was reported that Ad35 is an oncolytic virus. Ad35 was not inhibited by Ad5 antibodies, which many adults have, and showed a high antitumor effect [65]. However, the physical potency of Ad35 is less than one tenth of that of Ad5 [65], and considering the current efficiency of production/purification, Ad35 is likely not superior to Ad5F35.

Onyx-015 is a genetically modified adenovirus engineered to grow only in p53-deficient cancer cells by deleting the E1B region [43,44]; however, this method is not effective for only propagating adenoviruses in cancer cells. Strategies using the promoters of tumour-specific genes are more effective. Inserting the promoter regions of genes that are highly active in cancer cells, such as human telomerase reverse transcriptase [66] and cyclooxygenase 2 [67], at positions that control the expression of genes involved in adenoviral replication allows the oncolytic virus to replicate only in cancer cells. In addition, especially for the development of adenovirus-based oncolytic viruses, because half of the population is immune to adenoviruses [68] and the effect of the virus is thought to decline in a short period, “arming” them with therapeutic genes to enhance antitumour efficacy is also important [53]. The therapeutic genes being investigated for addition into oncolytic viruses include tumour suppressor (p53 and p16), cytokine-inducible [69,70], and immune stimulator genes [71]. Adenovirus-based oncolytic viruses have the advantage of being theoretically capable of “heavy-arming” with multiple therapeutic genes owing to their greater capacity limitations compared with that of other oncolytic viruses. 

Ad5-yCD/mutTKSR39rep-ADP is one of “heavy-arming” oncolytic viruses [72]. Yeast cytosine deaminase is catalyzes the deamination of the prodrug 5-fluorocytosine to form 5-fluorouracil [73]. Herpes simplex virus thymdine kinase is suicide gene in combination with ganciclovir [74]. ADP promotes the lysis and death of the host cell. At recent, the Ad5-yCD/mutTKSR39rep-ADP, second-generation, replicative adenoviral vector that containing a yeast cytosine deaminase (yCD), mutantSR39 herpes simplex virus thymdine kinase and adenovirus death protein (ADP) gene is in phase I trial (NCT03281382).

## 5. Use of Adenoviruses for Bladder Cancer

We have been researching virus therapy using adenoviruses to develop new treat-ment methods for urinary system cancer. 

Cancer of the urinary system is intractable. Approximately 70% of bladder cancer cases are NMIBC, which often develops into recurrent tumors and progresses to a higher stage or grade [75]. Currently, Bacillus Calmette-Guérin therapy is considered the most effective treatment for NMIBC; however, its severe side effects cause a significant reduction in patient QOL [76]. Nadofaragene firadenovec has already been approved in the US for gene therapy of NMIBC [36], and NMIBC is one of the subjects of the clinical trial CG0070 [53]. Renal cell carcinoma (RCC) accounts for over 90% of all kidney cancer cases [77]. Chemotherapy and molecular immunotherapy are not remarkably effective for RCC [78,79] and surgery is the most common treatment. However, surgery is difficult in some cases. Therefore, we focused on adenoviruses to develop new treatments for these cancers, designed chimeric adenoviral vectors, and examined their efficacies. 

**Figure 3:**
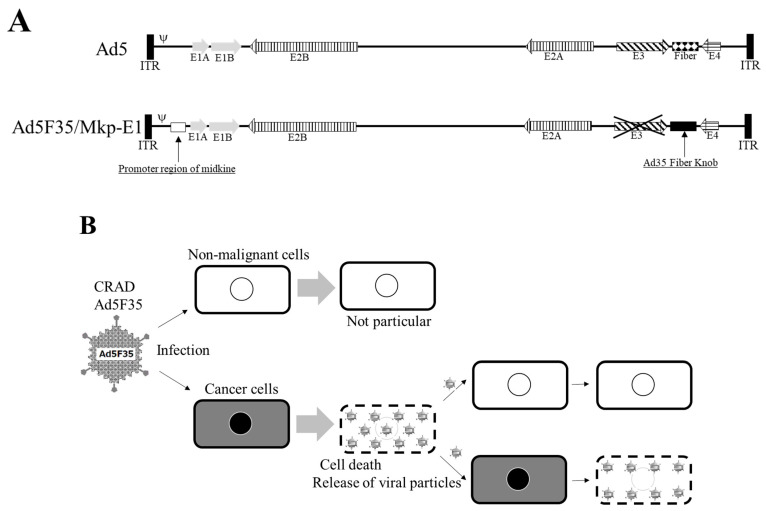
Combination of a chimeric adenovirus vector and midkine promoter. Because cluster of differentiation 46 (CD46) is also expressed in non-malignant cells, a safety device is required to prevent gene expression in these cells even if they are infected with an adenovirus serotype 5 F35 (AD5F35) vector. Safety is ensured by a conditionally replicating adenovirus (CRAD) that regulates genes necessary for adenovirus replication in the promoter region of midkine (**A**). The CRAD Ad5F35 vector as an oncolytic virus exhibits a remarkably potent oncolytic effect on cancer cells neighbouring primarily infected cells through secondary infection and replication (**B**). (**A**) is adapted from the data published by Nagaya et al., Anticancer Res, 2012 [95].

The safety of first-generation adenoviral vectors has been confirmed in clinical trials; however, relatively high inflammation caused by adenoviruses remains a major concern. Moreover, the major problem with cancer virotherapy using adenoviral vectors is the limited efficacy of gene transfer or the oncolytic effect because of lower CAR expression in highly malignant cancer cells [54,55,80]. Ad5F35 infects via CD46, but CD46 is expressed in almost all human cells; thus, its side effects on non-malignant tissues should be prevented. The conditionally replicating adenovirus (CRAD) vector is an effective solution for reducing the side effects [81]. Therefore, we devised a method to control adenovirus vectors using the promoter regions of proteins abundantly expressed in cancer cells. Midkine is a heparin-binding growth factor induced by retinoic acid in embryonal carcinoma cells [82] and is involved in mitogenesis, angiogenesis, anti-apoptosis, fibrinolysis, and transformation [83,84,85,86,87]. Midkine expression is enhanced in several cancers [88,89,90], whereas its expression in non-malignant cells is relatively limited. Thus, the promoter region [91] of the midkine gene (Figure 3A) can be used to control gene expression required for adenovirus replication to restrict the expression of introduced genes to cancer cells only. Therefore, we constructed an Ad5F35-Mkp-E1 CRAD vector encoding E1 under the control of a midkine promoter (Mkp; Figure 3A). The CRAD Ad5F35-Mkp-E1 vector, as an oncolytic virus, exhibited a significantly potent oncolytic effect on cancer cells neighbouring the primarily infected cells through secondary infection and replication (Figure 3B).

**Figure 4:**
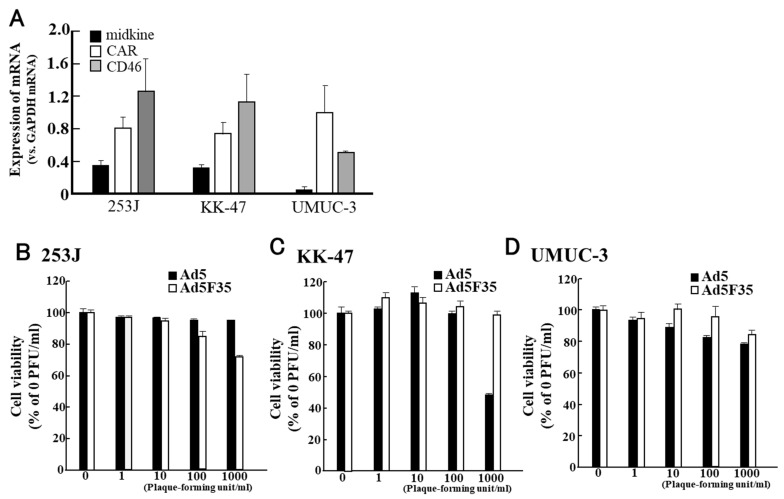
Expression of midkine, coxsackie and adenovirus receptor (CAR), and cluster of differentiation 46 (CD46) in human bladder cancer cell lines (**A**) and the oncolytic effects of adenovirus serotype 5 F35 (Ad5F35)/Mkp-E1 against bladder cancer cell lines (**B**–**D**). This figure is adapted from the data published by Gotoh et al., Urology, 2013 [92].

We examined the oncolytic effect of Ad5F35/Mkp-E1 in various cancer cell lines [92,93,94,95,96]. In bladder cancer cell lines (Figure 4) [92], midkine mRNA expression was observed in all experimental cell lines, with the lowest expression observed in UMUC-3 cells (Figure 4A). In 253J and KK47, CAR mRNA expression was considerably lower than that of CD46 mRNA (Figure 4A). In contrast, CAR mRNA expression in UMUC-3 was higher than that of CD46. In the 253J cell line, Ad5F35/MKp-E1 reduced cell viability in a PFU-dependent manner (Figure 4B), but no statistically significant difference was observed when compared to the antitumour effect of Ad5. Conversely, Ad5F35/Mkp-E1 had almost no effect on KK47 cells, even though the expression of CD46 mRNA was higher than that of CAR mRNA (Figure 4C). In UMUC3 cells, the expression of CAR mRNA was lower than that of CD46 (Figure 4A), and Ad5F35/Mkp-E1 was less effective in suppressing viability (Figure 4D).

These results suggested that the oncolytic effect of Ad5F35/MKp-E1 on human bladder cancer cells depends not only on the expression level of CD46 mRNA, but also on some Ad5F35/MKp-E1-targeted apoptosis-related molecules.

**Figure 5:**
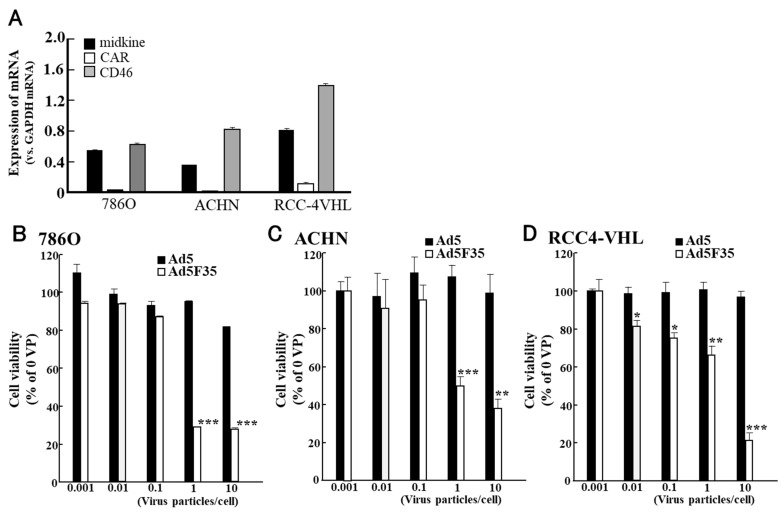
Coxsackie and adenovirus receptor (CAR) and cluster of differentiation 46 (CD46) expressions in renal cell carcinoma (RCC) cell lines (**A**) and the oncolytic effects of Ad5F35/Mkp-E1 against these cells (**B**–**D**). Only low CAR mRNA expression was found, and Ad5F35/Mkp-E1 reduced the cell viability of RCC cell lines. However, little or no effect was observed with Ad5/Mkp-E1 (**B**–**D**). This figure is adapted from the data published by Nagaya et al., Anticancer Res. 2012 [95]. *p* < 0.05: *, *p* < 0.01: **, *p* < 0.001: ***.

Unlike in bladder cancer cell lines, only low CAR mRNA expression was observed in RCC cell lines (Figure 5). In contrast, CD46 mRNA expression was significantly higher than that of CAR mRNA (Figure 5A). When comparing antitumour effects, only Ad5F35/Mkp-E1 reduced cell viability in RCC cell lines; however, little or no effect was observed with Ad5/Mkp-E1 (Figure 5B–D). These results demonstrated the ineffectiveness of conventional adenoviruses against RCC and the efficacy of Ad5F35 [95].

The combination of chimeric Ad5F35 and the Mkp can be applied for the development of oncolytic viruses and gene delivery vectors that express therapeutic genes in a cancer cell-specific manner.

**Figure 6:**
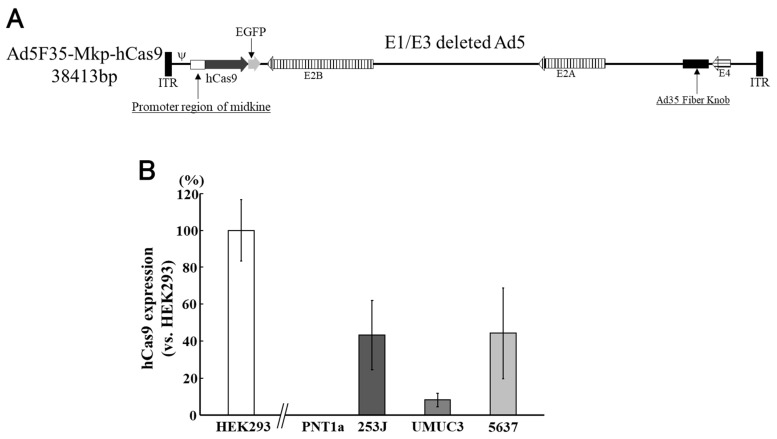
Human codon-optimised *Streptococcus pyogenes* Cas9 gene (*hCas9*) was placed downstream of the midkine promoter (Mkp) and the enhanced green fluorescent protein (*EGFP*) gene inserted next to it as an expression marker (**A**). In PNT1A, which is a non-malignant cell line, hCas9 expression was inhibited. In the bladder cancer cells lines, hCas9 protein expression was confirmed after Ad5F35-Mkp-hCas9 infection (**B**). This figure is adapted from the data published by Matsunaga et al., Anticancer Res. 2021 [99].

Clustered regularly interspaced short palindromic repeat-associated protein 9 (CRISPR-Cas9) technology is a versatile gene-editing tool with excellent clinical potential in the field of cancer therapy [96,97]. However, off-target effects of the CRISPR-Cas9 system are a major concern for its clinical application [98]. Therefore, we constructed a chimeric Ad5F35 vector containing Cas9, which is regulated by Mkp, to restrict gene expression in cancer cells. The upstream regulatory region of the midkine gene was used as a promoter. The human codon-optimised *Streptococcus pyogenes* Cas9 gene (*hCas9*) was placed downstream of Mkp, and an enhanced green fluorescent protein gene inserted next to it as an expression marker (Figure 6A) [99]. In PNT1A cells, a non-malignant cell line, hCas9 was not expressed, even in cells infected with Ad5F35-Mkp-hCas9. On the other hand, in bladder cancer cell lines, hCas9 protein expression was confirmed after Ad5F35-Mkp-hCas9 infection (Figure 6B). CD46 and midkine expression in bladder cancer cell lines have already been confirmed [92].

## 6. Discussion

Adenoviral vectors, including oncolytic viruses, are now considered relatively “old-fashioned”, but they still have the largest share of vectors used in clinical trials. Their high gene transfer efficiency, ease of manufacturing, and large cargo capacity are major advantages over the “newcomer” vectors. Although their high cytotoxicity is a drawback, the use of adenoviruses as oncolytic viruses is advantageous. The disadvantages of adenoviruses include their high immunogenicity and transient gene expression. However, this is also advantageous because overgrowth of oncolytic adenoviral vectors and aberrant gene expression are prevented. In addition, because several types of viruses that infect humans have been discovered, adenoviruses that are more suitable as vectors than Ad5 are also expected to be discovered. Therefore, future studies on adenoviral vectors for viral therapy should be conducted.

However, Japan lags behind other developed countries when it comes to the development of gene therapies, including studies using viral vectors. Studies in Japan account for only 1.5% of the clinical gene therapy trials conducted worldwide [3].

In Japan, the government regulations for gene therapy studies are strict. When initiating clinical studies on gene therapy in Japan, the Clinical Research Act requires examination and reporting to the Ministry of Health, Labour, and Welfare. Moreover, a review by a committee stipulated in the “guidelines about the clinical study including gene therapies” that approval by the Minister of Health, Labour, and Welfare is also required. The Cartagena Protocol on Biosafety does not regulate medical studies. However, the Japanese law that corresponds to the protocol, the so-called “Cartagena Act”, regulates medical studies, and clinical studies on gene therapy require approval from the minister in charge. These regulations involve complex and time-consuming procedures. We aimed to conduct clinical studies using adenoviruses in Japan. However, unless strict regulations are changed, the development of gene therapy studies will be hindered, regardless of whether adenovirus vectors are used. We hope that the Japanese government will promptly ease the restrictions on gene therapy studies.

## Figures and Tables

**Table 1:** Changes in vectors used in gene therapy clinical trials over the past decade.

	Share in Clinical Trials until 2012 [5]		Share in Clinical Trials until 2017 [6]		Share in Clinical Trials until 2022 [3]		Increase 2012 to 17	Increase 2017 to 22
Adenovirus	23.3%	438	20.5%	547	15.5%	573	109	26
Retrovirus	19.7%	370	17.9%	478	14.6%	538	108	60
Plasmid DNA	18.3%	345	16.6%	442	13.1%	483	97	41
Lentivirus	2.9%	55	7.3%	196	9.9%	364	141	168
AAV	4.9%	92	7.6%	204	9.5%	350	112	146

AAV: adeno-associated virus.

**Table 2:** Characteristics of the top five gene vectors used in gene therapy clinical trials.

	Nondividing Cell	Cargo Limit	Duration of Gene Expression	Physical Containment	Immunogenicity of Vectors	Drawbacks	Safety
Adenovirus	Yes	7–36 kb	Transient *	P2	High	Strong cytotoxicity	Moderate
Retrovirus	No	8–9 kb	Stable	P2	Moderate	Risk of the carcinogenesis	Moderate
Plasmid DNA	Yes	limitless	Transient	-	-	Low transduction efficiency	High
Lentivirus	Yes	8–9 kb	Stable	P2	Moderate	Concerns about genes derived from HIV-1	High
AAV	Yes	4.7 kb	Potentially Stable	P1	Low	Difficult to purification, Small cargo limit	High

*: Third-generation adenoviral vectors are “stable” under certain conditions. AAV: adeno-associated virus.

## Data Availability

No new data were created or analyzed in this study. Data sharing is not applicable to this article.
